# Regucalcin ameliorates doxorubicin-induced cytotoxicity in Cos-7 kidney cells and translocates from the nucleus to the mitochondria

**DOI:** 10.1042/BSR20211464

**Published:** 2022-01-06

**Authors:** Noor A. Mohammed, Israa J. Hakeem, Nikolas Hodges, Francesco Michelangeli

**Affiliations:** 1 School of Biosciences, University of Birmingham, Edgbaston, Birmingham, B15 2TT, U.K.; 2 Department of Biology, College of Science, University of Duhok, Zakho Street 38, 1006 AJ, Duhok, PO Box 78, Iraq; 3 Faculty of Medicine, Dentistry and Life Sciences, University of Chester, Parkgate Rd, Chester, CH1 4BJ, U.K.

**Keywords:** apoptosis, calcium signalling, colocalization, necrosis, regucalcin

## Abstract

Doxorubicin (DOX) is a potent anticancer drug, which can have unwanted side-effects such as cardiac and kidney toxicity. A detailed investigation was undertaken of the acute cytotoxic mechanisms of DOX on kidney cells, using Cos-7 cells as kidney cell model. Cos-7 cells were exposed to DOX for a period of 24 h over a range of concentrations, and the LC_50_ was determined to be 7 µM. Further investigations showed that cell death was mainly via apoptosis involving Ca^2+^ and caspase 9, in addition to autophagy. Regucalcin (RGN), a cytoprotective protein found mainly in liver and kidney tissues, was overexpressed in Cos-7 cells and shown to protect against DOX-induced cell death. Subcellular localization studies in Cos-7 cells showed RGN to be strongly correlated with the nucleus. However, upon treatment with DOX for 4 h, which induced membrane blebbing in some cells, the localization appeared to be correlated more with the mitochondria in these cells. It is yet to be determined whether this translocation is part of the cytoprotective mechanism or a consequence of chemically induced cell stress.

## Introduction

Doxorubicin (DOX) is a potent anticancer drug, which consists of an amino-sugar daunosamine bound to a tetracycline moiety [1]. Biochemically, DOX reacts and interferes with DNA and DNA polymerases at G1 and G2 phases of the cell cycle, inhibiting replication [2]. In addition, DOX has been shown to affect mitochondrial function and cause elevation of reactive oxygen species [3]. DOX has also been shown to decrease the anti-apoptotic factor Bcl2 and increase the pro-apoptotic factor Bax, which causes cytochrome *c* release from the mitochondria and leads to apoptosis via caspases 9 and 3 activation [4].

Nephrotoxicity is one of the more notable side-effects of anthracycline antibiotics such as DOX [1,5] and can therefore limit its uses in some patients that show renal impairment. As such an investigation into its mechanisms of cytotoxicity in kidney cells, as well as identifying approaches that can reduce this nephrotoxicity, would be of potential clinical relevance.

Regucalcin (RGN) is a 299 amino acid polypeptide (33 kDa MW) which is found inside the cells [6,7]. RGN is a multifunctional protein that is involved in a number of cellular processes including calcium homeostasis by regulating Ca^2+^-binding protein activity such as Ca^2+^ ATPases, calmodulin kinase and PKC [8]. Furthermore, RGN has an established defensive role in Ca^2+^-mediated cell stress [8,9]. RGN has also been shown to be an important ageing biomarker in many organisms, as its expression increases through the neonatal stage into early adulthood and then decreases significantly in old age [10,11]. Therefore, RGN is also commonly referred to as Senescence Marker Protein 30 (SMP30) [10–12].

Subcellular localization studies of RGN have shown it to be mainly located in the cytosol and nucleus [8,9,13] and to a lesser extent in the mitochondria [13,14], and it is predominately found in tissues such as kidney, liver and brain [15].

Overexpression of RGN decreases liver cell death when cells are treated with agents such as PD89059 and dibucaine [16]. Increased expression of RGN has also been shown to reduce the level of cell death caused by the calcium pump inhibitor and ER stress inducer, thapsigargin [16,17]. Furthermore, RGN has also been shown to cause a rise in the anti-apoptotic factor Bcl2 expression in NRK52E kidney cells [18].

In the present study, we investigate the mechanism of cytotoxicity for DOX on Cos-7 kidney cells and assess the effects of RGN expression on this process.

## Materials and methods

### Cell culture

Cos-7 cells were grown in Dulbecco’s modified Eagle’s medium (DMEM) supplemented with 2 mM L-glutamine, penicillin (20 units/ml), streptomycin (20 mg/ml), 1% non-essential amino acids and containing 10% (vol/vol) heat-inactivated fetal bovine serum (FBS). Cells were maintained at 37°C in a saturated humidity atmosphere containing 95% air and 5% CO_2_.

### Cell viability studies

Cells were seeded in 24-well plates and allowed to reach 80–90% confluency at 37°C. Different concentrations of DOX were incubated with the cells for 24 h. Cell viability was measured by MTT assays. Cells were incubated for about 60 min in MTT solution made in Hank’s buffer (0.5 mg/ml, final concentration), and after removing the MTT solution, the cells were dissolved in 1 ml DMSO. The solubilized cell suspension was moved to 96-well plate and absorbance measured using an ELISA plate reader at 590 nm [19].

In some experiments, the cells were pre-treated with either: BAPTA-am (10 µM) (a membrane permeable Ca^2+^ chelator); 50 µM caspase 3 inhibitor (Ac-DEVD-cmk); 50 µM caspase 9 inhibitor (Z-LEHD-fmk); or 10µM necrostatin-1, for typically 4 hours prior to treatment with DOX.

For lactate dehydrogenase (LDH) activity measurements to assess necrosis, the cells were seeded in to 6-well plates overnight; the following day, the drugs were added to the wells and left for 24 h, following which the media surrounding the cells were collected and centrifuged at 1, 500 rpm for 5 min. In some wells, 1% Triton X-100 was also added and incubated for 30 min (100%, positive control). About 50 µl of each sample was added to 1 ml assay buffer (100 mM potassium phosphate, 0.66 mM sodium pyruvate, 0.25 M NADH pH 7), and the rate of oxidation of NADH was followed at 340 nm in spectrophotometer.

### RGN transfection

Overexpression of RGN was via transfection of Cos-7 cells using the plasmids for Human RGN cDNA clone (Pcmv6 AC-GFP-RGN with C-terminal GFP tag) from Origene or human RGN inserted into pcDNA 3.1- (as used in [20]). Turbofect (ThermoFisher) was used to transfect the cells, following the manufacturer’s guidelines. To monitor autophagy the cells were transfected as above with a GFP-LC3 containing plasmid which was supplied by Prof. Vincent Wong (University of Macau). Cells were deemed positive for autophagy if they showed several clearly defined fluorescent puncta, indicative of autophagosomes within the body of the cells [21].

### Western blotting

Western blotting was as described previously in [22] with the following modifications: Lysates were prepared by adding lysis buffer (50 mM Tris-HCl, 150 mM NaCl, 1 mM EDTA, 1% Triton X100, 1% sodium deoxycholate, 0.1% SDS and 1 mM PMSF, pH 7.4) to untransfected Cos-7 or RGN transfected cells attached to the surface of wells in a 12-well plate. A lysate from rat liver homogenate was also prepared as a positive control. The RGN antibody as used in [20] was diluted at 1:50 for anti-RGN.

### Co-localization studies

For co-localization studies of RGN, Cos-7 cells were seeded on to sterilized coverslips in 6-well plates overnight at 37°C. The cells were transfected with RGN-GFP in 2ml media (serum free DMEM) and viewed under the epi-fluorescence microscope after 48 h. Cells were then treated with DOX (20 µM) in 2 ml serum-free media for 4 h. Cells were washed with PBS three times and then treated with (0.5 µM) Mitotracker® Deep RedFM in 2 ml DMEM media and incubated for 30–40 min. Cells then were washed with PBS, two times and then the DMEM media replaced by 4% paraformaldehyde (PFA) in PBS pH 7.4 to allow the cells to be fixed. After 20 min, cells were washed with PBS twice and 1 µg/ml of Hoechst 33258 was added to the cells, which were then covered in foil and incubated for a further 30 min. Finally, cells were washed again with PBS twice more. The coverslips were removed and 70 µl of Hypomount agar was added to the sides of the coverslip and left in the dark to allow air drying to occur. The stained and fixed cells were viewed with either, white light, or the Red, Blue and Green channels of either a Leica SP2 or Nikon Eclipse Ti microscope, set up for epi-fluorescence or bright-field imaging.

### Statistical analysis

The analysis of the co-localization was undertaken using the JACOps application in ImageJ [23], which compares the pixel intensities for two of the fluorophores at each location on the micrograph and calculates the Pearson’s correlation coefficient (which can be used as a measure of co-localization) [23]. For changes in cell viability studies the data were analyzed by unpaired two-tailed Student *T*-tests and *P* values of less than 0.05 was considered to be significant. For some of the data where more than one variable was being compared a one-way ANOVA was undertaken.

## Results

### Cytotoxicity of DOX on Cos-7 cells

**Figure 1:**
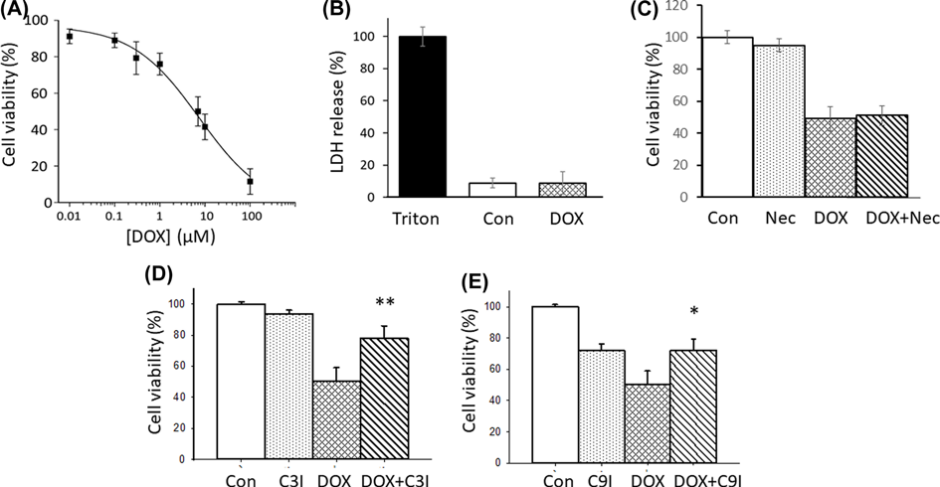
Effects of Doxorubicin on cell viability and cell death mechanisms Panel (**A**) shows the effects of doxorubicin on cell viability of Cos-7 cells following 24 h exposure. Panel (**B**) shows the effects of DOX (7 µM) on the leakage of LDH from Cos-7 cells after 24 h of exposure. Treatment with 1% triton was used as a positive control to induce complete leakage of LDH from cells. Panel (**C**) shows the effects of 4 h pre-treatment with necrostatin-1 (10 µM) upon cell viability of Cos-7 cells then exposed to DOX (7 µM) for 24 h. Panel (**D**) shows the effects of 4 h pre-treatment with caspase 3 inhibitor (50 µM) upon cell viability of Cos-7 cells that were then exposed to DOX (7 µM) for 24 h. Panel (**E**) shows the effects of 4 h pre-treatment with caspase 9 inhibitor (50 µM) upon cell viability of Cos-7 cells that were then exposed to Dox (7 µM) for 24 h. All data are presented as the mean ± SD of four replicates. Statistical analysis via two-tailed Student *T*-test was performed in comparing cell viability levels in DOX-treated cells in the presence and absence of caspase inhibitors. Probability values *P<0.05, **P<0.01.

Figure 1A shows the effects of DOX on cell viability of Cos-7 cells exposed for a period of 24 h. The LC_50_ value was determine to be 7 ± 1 µM. This indicates that DOX is extremely potent at inducing cell death as its LC_50_ value was considerably lower than other commonly used anti-cancer drugs such as cisplatin or etoposide which had LC_50_ values in ≈50–150 µM concentration range for Cos-7 cells (data not shown). In order to assess whether DOX induces necrosis, LDH release form Cos-7 cells treated with DOX was undertaken. Figure 1B shows that similar, low levels of LDH release, was observed from cells in the presence or absence of DOX (7 µM) treated for 24 h, indicating that membrane integrity was unaffected, especially when compared with cells treated with triton X-100. In order to determine whether regulated necrosis (necroptosis) played a role in inducing DOX-mediated cell death, the cells were pre-incubated with for 4 h with 10 µM necrostatin-1 (Nec; an inhibitor of RIPK 1/2). Again, no difference in the number of viable cells was observed when comparing Nec-pretreated cells with non-pretreated cells that were subsequently exposed for 24 h in either the presence or absence of DOX (Figure 1C). Furthermore, pre-treating the cells with either the caspase 3 inhibitor (C3I) or the caspase 9 inhibitor (C9I) before the cells were then exposed to 7 µM DOX, also greatly increased the percentage of viable cells (Figure 1D,E), thus supporting a major role for apoptosis in DOX-induced cell death.

**Figure 2:**
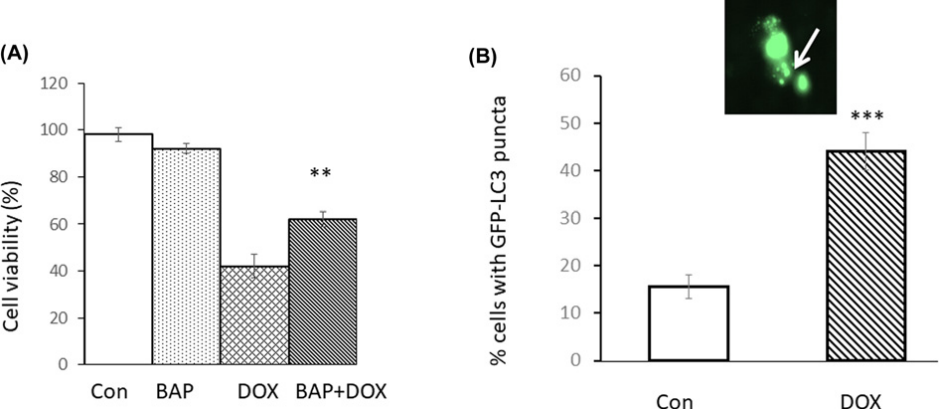
Doxorubicin causes Ca^2+^-dependent cell death and autophagosome formation Panel (**A**) shows the effects of 4 h pre-treatment with the Ca^2+^ chelator BAPTA-am (10 µM) upon cell viability of Cos-7 cells that were then exposed to DOX (7 µM) for 24 h. Data are presented as the mean ± SD of four replicates. (**B**) Cells were transfected with GFP-LC3 for 48 h and then treated with 7 µM DOX for 4 h prior to analysis by fluorescence microscopy. Inset shows a cell with clear puncta indictive of autophagosome formation. The experiment was repeated in triplicate and SD determined of fluorescent cells that clearly showed the presence of puncta either in the presence or absence of DOX. Statistical analysis via two-tailed Student’s *T*-test was performed in comparing cell viability levels in DOX-treated cells in the presence and absence of BAPTA-am, or % cells showing GFP-LC3 puncta when treated in the absence or presence of DOX. Probability values **P<0.01, ***P<0.005.

In order to determine whether apoptotic cell death was, at least in part, due to a Ca^2+^-mediated mechanism, the cells were pre-treated with the intracellular Ca^2+^ chelating agent, BAPTA-am (BAP) for 5 h prior to exposure with DOX. Figure 2A showed that BAPTA was able to significantly reduce cell death, indicating a Ca^2+^-mediated mechanism is at least partly involved. Figure 2B shows that treatment with DOX (7 µM) for 4 h increased the percentage of cells with clearly defined fluorescent puncta indicating the formation of autophagosomes by almost 3-fold compared with control cells.

### Expression of RGN and RGN-GFP and effects on DOX toxicity

**Figure 3:**
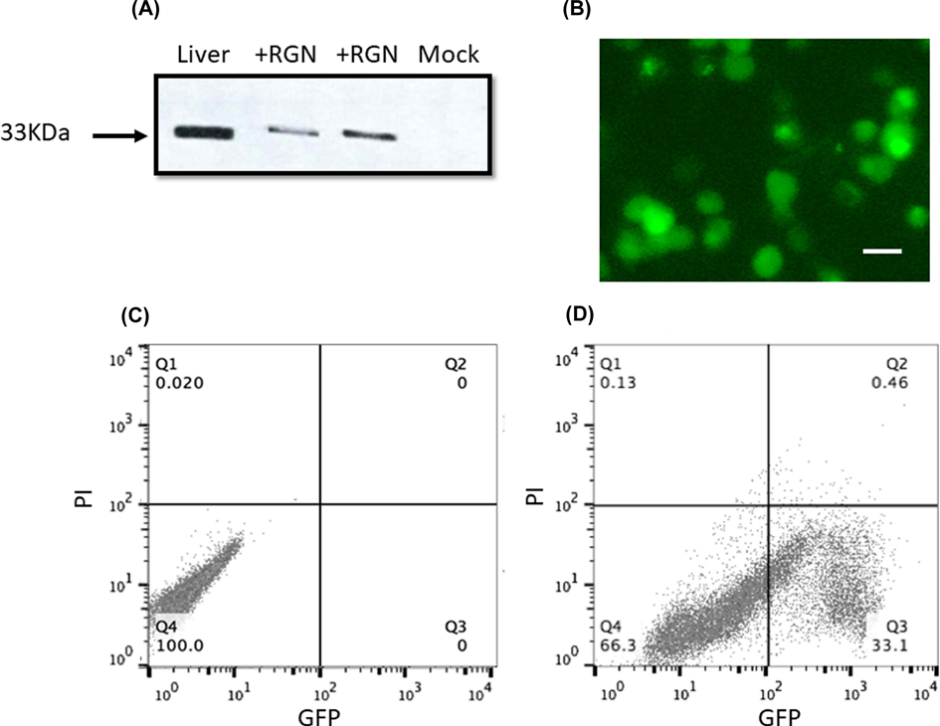
Expression of Regucalcin in Cos-7 cells Panel (**A**) shows a Western blot of lyasates of: mock transfected Cos-7 cells; RGN transfected cells and rat liver homogenate, as a positive control. The lanes were loaded with 7.5μg of protein and probed with a RGN polyclonal antibody (1:50 dilution). A protein labeled band at approximately 33kDa was observed in RGN-transfected lysate and liver homogenate. The Image was modified for clarity by changing the brightness and contrast as well as some smoothing. Panel (**B**) shows a fluorescence micrograph of Cos-7 cells transfected with Pcmv6 AC-GFP-RGN, indicating the presence GFP-RGN expressing cells. The white bar indicates 20 µm. (**C**) Untransfected Cos-7 cells analyzed by flow-cytometry, monitoring both the red (propidium iodide) and green (GFP) channels. The untransfected cells were considered to be fully viable, as all cells were binned within quadrant Q4 (i.e. minimal levels of propidium iodide staining). Panel (**D**) shows RGN-GFP transfected cells analyses by flow cytometry. The cells were again considered to be fully viable as they were binned within quadrants Q4 and Q3 (minimal levels of PI staining). From the analysis of the green fluorescence channel (GFP) more than 33% of the cells analyzed expressed GFP.

Cos-7 cells were transfected with mammalian expression plasmids that encoded either the human variant 1 for RGN or regucalcin-c-tagged with GFP (RGN-GFP). Figure 3A,B shows that both RGN and RGN-GFP were successfully transfected into Cos-7 cells with a transfection efficiency for the RGN-GFP of between 30 and 45% as determined by comparing the same cells within a defined field of view, when viewed under bright-field illumination and then in the green channel in epi-fluorescence mode (Figure 3D) [20]. In addition, transfection with RGN-GFP was found to have little effects upon cell viability as only a small percentage of cells were considered to be non-viable compared to untransfected cells as determined by propidium iodide versus GFP fluorescence measurement using flow cytometry (Figure 3C,D).

**Figure 4:**
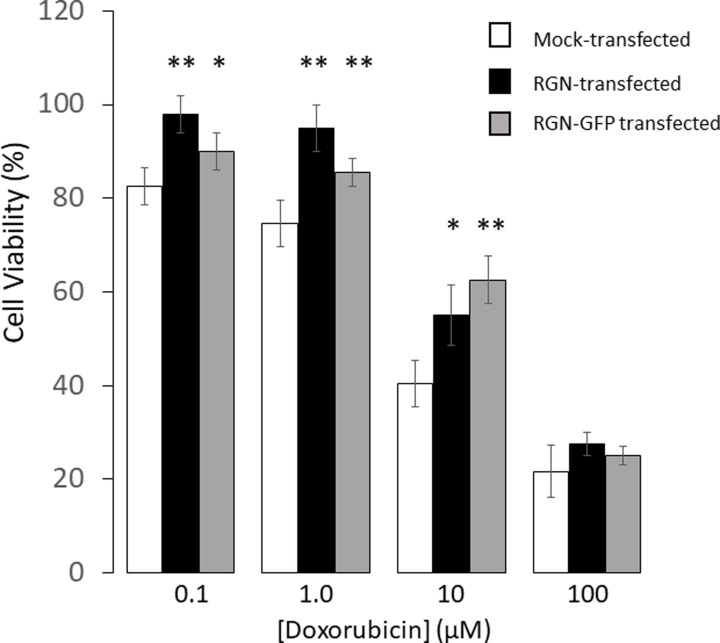
Effects of RGN expression on the cell viability in the presence of Doxorubicin Cos-7 cells were either mock-transfected with empty plasmid (white) or transfected with RGN (black) or RGN-GFP (gray) plasmid for 48 h. The cells were then treated with DOX over a range of 0 to 100 μM concentration for a period of 24 h. Cell viability was then monitored by MTT assays. The data represent the mean ± SD of 4 determination. Statistical analysis via two-tailed Student *T*-test was performed in comparing cell viability levels in DOX treated cells that were either mock-transfected or transfected with either RGN or RGN-GFP. Probability values *P<0.05, **P<0.01.

MTT cell viability measurements were performed with RGN-, RGN-GFP-transfected and mock-transfected Cos-7 cells following 24 h exposure with a range of concentrations of DOX. Figure 4 shows that over a range of DOX concentrations, the percentage of viable cells in the mock-transfected group were reduced more than the cells transfected with either RGN or RGN-GFP. In mock-transfected cells the LC_50_ for DOX was about 7 µM, similar to that observed in untransfected Cos-7 cells (Figure 1A). For both RGN and RGN-GFP transfected Cos-7 cells the LC_50_ values increased to approximately 20 µM. These results demonstrate that increasing the expression of RGN ameliorates the toxicity of Cos-7 cells to DOX. Furthermore, a one-way ANOVA showed a statistically non-significant difference in the levels of viable cells when comparing RGN-transfected cells and RGN-GFP-transfected cells, over a range of DOX concentrations.

### Localization of RGN-GFP

**Figure 5:**
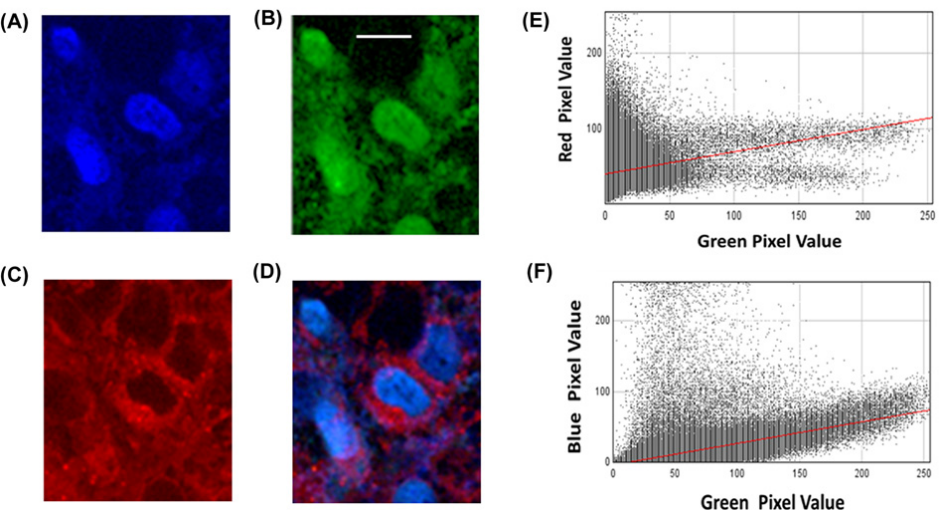
Subcellular localization of RGN-GFP in Cos-7 cells Cos-7 cells transfected with RGN-GFP (green) were stained with mito-tracker (red) and Hoechst (blue) and then fixed with paraformaldehyde. The fixed cells were then viewed under a fluorescence microscope under (**A**) blue (nuclei), (**B**) green (RGN-GFP) channels and (**C**) red (mitochondria), to discriminate between the locations. Panel (**D**) shows the superimposition of all three channels. Panel (**E**) shows the scatter plot of individual pixel intensities within the image for the green and red channels. Panel (**F**) shows the scatter plot of individual pixel intensities within the image for the green and blue channels. The images are presentative of three separate experiments, each analyzing several fields of view. The Pearson’s correlation coefficient between RGN and mitochondria was determined to be 0.19 and for RGN and nuclei it was 0.56, when analyzed for the three experiments.

Previous studies using cell fractionation and immunolocalization of RGN in a variety of cell and tissue types has shown that it is mainly localised to the nucleus and cytoplasm with very little in the mitochondria [13,14,20]. Here, we have used RGN-GFP (visualized in green with GFP) to monitor its localization compared with the nucleus (visualized in blue with Hoechst) and mitochondria (visualized in red with Mitotracker Red). Figure 5 shows fluorescence micrographs of untreated transfected cells imaged under different spectral conditions in order to identify the presence of nuclei (blue) (Figure 5A), RGN-GFP (green) (Figure 5B) and mitochondria (red) (Figure 5C) within cells. Visual analysis of the micrographs showed a much more evident superimposition between RGN-GFP and nuclei than between RGN-GFP and mitochondria (i.e. compare Figure 5A versus 5B and compare Figure 5B versus 5C). More detailed analysis of the co-localization using the JACOps plugin within ImageJ, to calculate the Pearson’s correlation coefficient for overlap between the two fluorophores, showed that in untreated cells the Pearson’s correlation coefficient between RGN-GFP and mitochondria (Figure 5E) was determined to be 0.19 ± 0.07, while the coefficient for RGN-GFP and nuclei (Figure 5F) was considerably higher at 0.56 ± 0.04, which supported previous studies that RGN is substantially co-localized to the nucleus [13].

**Figure 6:**
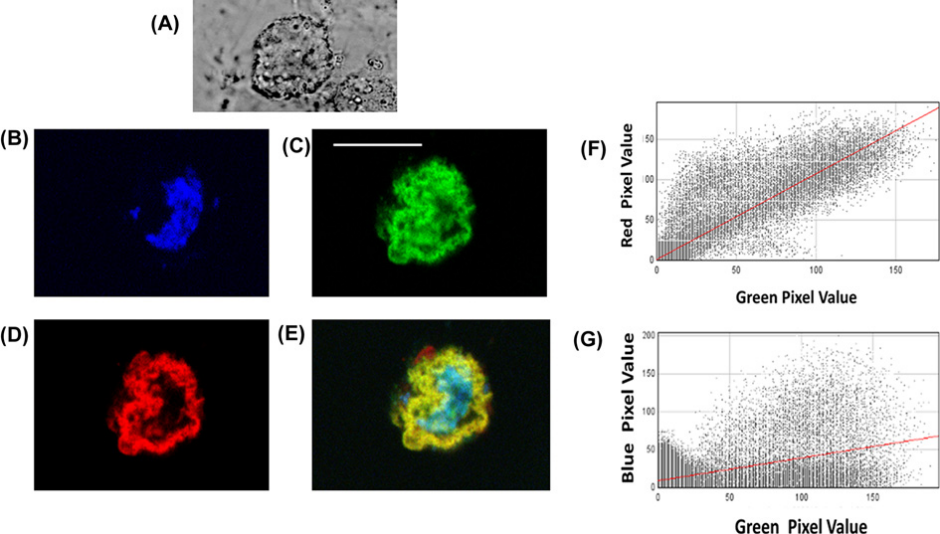
Subcellular localization of RGN-GFP in Cos-7 cells treated with Doxorubicin Cos-7 cells transfected with RGN-GFP (green) were then treated with DOX (20 μM) for 4 h before being stained with mito-tracker (red) and Hoechst (blue) and then fixed with paraformaldehyde. The fixed cells were then viewed under a fluorescence microscope under (**A**) bright field illumination, to show morphological changes; (**B**) blue (nuclei); (**C**) green (RGN-GFP) channels and (**D**) red (mitochondria), to discriminate between the different locations. Panel (**E**) shows the superimposition of all three channels. Panel (**F**) shows the scatter plot of individual pixel intensities within the image for the green and red channels. Panel (**G**) shows the scatter plot of individual pixel intensities within the image for the green and blue channels. The images are presentative of three separate experiments, each analyzing several fields of view. The Pearson’s correlation coefficient between RGN and mitochondria was determined to be 0.8 and for RGN and nuclei it was 0.41, when analyzed for the three experiments.

RGN-GFP transfected Cos-7 cells were then imaged following 4 h treatment with 20 µM DOX (Figure 6A–D). A number of the cells were showing visible signs of stress such as shape change and membrane ruffling/blebbing (Figure 6A). Upon visual inspection of these cells it was clear that the location of the RGN-GFP had altered, becoming much more co-localized with the mitochondria (Figure 6B–E). This was also confirmed by determination of the Pearson’s correlation coefficient which was 0.41 ± 0.05 for co-localization between RGN-GFP and nuclei (Figure 6F) and 0.80 ± 0.08 between RGN-GFP and mitochondria (Figure 6G).

## Discussion

DOX is an anthracycline antibiotic anticancer drug which is considered to be one of the most potent chemotherapeutic drugs that is currently used [1]. This drug has the ability to damage DNA and inhibit DNA and RNA production by free-radicals damage [24]. In the present study, we found that cell death of Cos-7 kidney cells by DOX mainly occurred through apoptosis. This finding is consistent with other studies that have shown DOX to induce apoptosis in renal cells [25], hepatoma cells [26] and neuroblastoma cells [27]. From the results shown in this study, cell death by DOX was unlikely to involve necrosis as little loss of membrane integrity was observed as assessed from LDH release assays (Figure 1B). This disagrees with the reports of DNA damage-induced necrosis through a PARP1-dependent and p53-independent pathway by DOX in HK2 cells [28]. As no effects were seen for either the LDH release assay or in the presence of necrostatin inhibitor, an inhibitor of regulated necrosis (Figure 1B), we believe that different; cell lines, experimental conditions and treatment times (we used acute 24 hours exposure of the cells), may account for some of these differences.

Cos-7 cells overexpressing RGN, with or without the GFP tag, had the ability to decrease the toxicity of DOX. From the results presented in Figures 1 and 2 which show cell protective effects of both caspase inhibitors (3 and 9), as well as BAPTA-am, we infer DOX is inducing cell death through Ca^2+^-mediated apoptosis involving caspase 9. Furthermore, expression of RGN appears to substantially ameliorate DOX-induced cell death (Figure 4). In addition, another pathway that appears to be involved in DOX induced cell death was autophagy (Figure 2B) which we have previously indicated can also be activated through Ca^2+^-mediated processes [21,29]. This finding is supported by a recent study that also showed that the toxicity of DOX on cardiac cells involves autophagy [30].

Our findings that RGN reduces cell death by chemical agents are supported by other studies, which showed a suppressive effect of RGN on apoptosis in kidney cells treated with a range of factors such as TNF-α, LPS, Bay K 8644 and thapsigargin [18]. Accordingly, RGN has also been shown to have an important role in reducing apoptosis in cells through various signalling factors [8,9,31]. For instance, TNF-α increases the mRNA expression of caspase-3 in rat kidney proximal tubular epithelial cells and the overexpression of RGN within these cells appears to reduce this effect [31]. To further support a role of RGN in suppressing apoptosis, overexpression of RGN in H4-II-E hepatoma cells was found to have a suppressive effect on cell death induced by sulforaphane by affecting the levels of pro- and anti-apoptotic factors such as Bax, cytochrome *c* and Bcl-2, that directly control mitochondrial mediated apoptosis via caspase 9 [32].

The co-localization studies presented here have shown that, in untreated cells, RGN is mostly found within the nucleus, with limited co-localization to the mitochondria (Figure 5). However, Cos-7 cells treated with DOX showed a very significant co-localization with mitochondria, and a much more limited co-localization within nuclei (see Figure 6). This suggests that RGN is usually distributed mostly in nuclei under normal conditions as shown by other studies [13,20]; however, this protein appears to move to the mitochondria during the latter stage of cell death possible trying to protect cells by potentially altering mitochondrial mediated processes associated with caspase 9 activation. This observation appears to be the first reported case that RGN can translocate to the mitochondria during chemically-induced cell stress.

We have previously shown that RGN is able to increase the expression of sarcoplasmic endoplasmic Ca^2+^-ATPases (SERCA) within Cos-7 cells [20], and since we have shown that DOX induces cell death through a Ca^2+^-mediated apoptotic pathway, one possible role is that RGN suppresses both the exaggerated cytosolic Ca^2+^ elevation needed to trigger this pathway by both removing excess Ca^2+^ back to the ER, as well as into the mitochondria (which can also act as a Ca^2+^ storage organelle [33]), since RGN was also shown to increase Ca^2+^ uptake in mitochondria [6,34]. Furthermore, such a translocation of RGN to the mitochondria could explain, the fact that since DOX can also induce oxidative stress [3,35], RGN translocating to the mitochondria could be a mechanism by which it is trying to reduce this mitochondrially driven oxidative stress process [36]. However, further work is required to determine whether the translocation of RGN to the mitochondria is part of the protective mechanism in trying to reduce cell death, or a downstream consequence of the initiation of cell death.

One of the biggest issues with regards to DOX as an anticancer agent is its cardiotoxic and nephrotoxic side effects, which in some cases can lead to patients having to stop treatment. This could therefore have serious consequences to the long-term prognosis of these patients. Being able to protect against the adverse effects of DOX causing damage to kidney cells, possibly by increasing the levels of RGN, could be a possible strategy reducing this damage. Recent studies have shown that kidney cells and tissues can be induced to express relatively high levels of RGN by treating them to a range of hormones such as parathyroid hormone, aldosterone or dexamethasone [37]. If increasing RGN levels in patients could be achieved by means of administering these hormones prior to DOX chemotherapy, this might provide a means of minimising these nephrotoxic side effects. Further studies are therefore required to explore such possibilities.

## Conclusion

In summary, the present study has shown that DOX induces cell death in kidney derived Cos-7 cells through Ca^2+^-dependent apoptosis and autophagy. In addition, cell death by DOX is reduced when RGN is expressed within these cells. During DOX exposure, RGN also appears to translocate from the nucleus to the mitochondria, which may be contributing factor to its protective role.

## Data Availability

Data and detailed experimental procedures will be made available upon request to the corresponding author.

## Abbreviations

C3I: caspase 3 inhibitor
C9I: caspase 9 inhibitor
DOX: Doxorubicin
LDH: lactate dehydrogenase
Nec: necrostatin
PFA: paraformaldehyde
RGN: Regucalcin
SMP30: Senescence Marker Protein 30

## References

[1] Carvalho C., Santos R., Cardoso S., Correia S., Oliveira P., Santos M. et al. (2009) Doxorubicin: the good, the bad and the ugly effect. Curr. Med. Chem. 16, 3267–3285 10.2174/092986709788803312 19548866

[2] Lal S., Mahajan A., Chen W. and Chowbay B. (2010) Pharmacogenetics of target genes across doxorubicin disposition pathway: a review. Curr. Drug Metab. 11, 115–128 10.2174/138920010791110890 20302569

[3] Armstrong J. and Dass C. (2018) Doxorubicin action on mitochondria: relevance to osteosarcoma therapy? Curr. Drug Targets 19, 432–438 10.2174/1389450116666150416115852 25882220

[4] Leung L. and Wang T. (1999) Differential effects of chemotherapeutic agents on the Bcl-2/Bax apoptosis pathway in human breast cancer cell line MCF7. Breast Cancer Res. Treat. 55, 73–83 10.1023/A:1006190802590 10472781

[5] Ayla S., Seckin I., Tanriverdi G., Cengiz M., Eser M., Soner B.C. et al. (2011) Doxorubicin induced nephrotoxicity: protective effect of nicotinamide. Int. J. Cell Biol. 2011, 390238 10.1155/2011/390238 21789041 PMC3140777

[6] Omura M. and Yamaguchi M. (1999) Regulation of protein phosphatase activity by regucalcin localization in rat liver nuclei. J. Cell. Biochem. 75, 437–445 10.1002/(SICI)1097-4644(19991201)75:3<437::AID-JCB9>3.0.CO;2-W 10536367

[7] Yamaguchi M. and Yamamoto T. (1978) Purification of calcium binding substance from soluble fraction of normal rat liver. Chem. Pharmaceut. Bull. 26, 1915–1918 10.1248/cpb.26.1915 699201

[8] Yamaguchi M. (2005) Role of regucalcin in maintaining cell homeostasis and function (review). Int. J. Mol. Med. 15, 371–389 10.3892/ijmm.15.3.371 15702226

[9] Yamaguchi M. (2012) Regucalcin: Genomics, Cell Regulation and Diseases, Nova Science Publishers, New York

[10] Ishigami A., Masutomi H., Handa S. and Maruyama N. (2015) Age-associated decrease of senescence marker protein-30/gluconolactonase in individual mouse liver cells: immunohistochemistry and immunofluorescence. Geriatr. Gerontol. Int. 15, 804–810 10.1111/ggi.12347 25311772

[11] Fujita T., Shirasawa T., Uchida K. and Maruyama N. (1996) Gene regulation of senescence marker protein-30 (SMP30): coordinated up-regulation with tissue maturation and gradual down-regulation with aging. Mech. Ageing Dev. 87, 219–229 10.1016/0047-6374(96)01711-3 8794449

[12] Fujita T., Shirasawa T. and Maruyama N. (1996) Isolation and characterization of genomic and cDNA clones encoding mouse senescence marker protein-30 (SMP30). Biochim. Biophys. Acta 1308, 49–57 10.1016/0167-4781(96)00064-4 8765750

[13] Nakagawa T. and Yamauchi M. (2008) Nuclear localization of regucalcin is enhanced in culture with protein kinases C activation in cloned normal rat kidney proximal tubular epithelial NRK25E cells. Int. J. Mol. Med. 21, 605–610 10.3892/ijmm.21.5.605 18425353

[14] Yamaguchi M., Takakura Y. and Nakagawa T. (2008) Regucalcin increases Ca2+-ATPase activity in the mitochondria of brain tissues of normal and transgenic rats. J. Cell. Biochem. 104, 795–804 10.1002/jcb.21664 18181158

[15] Yamaguchi M. and Isogai M. (1993) Tissue concentration of calcium-binding protein regucalcin in rats by enzyme-linked immunoadsorbent assay. Mol. Cell. Biochem. 122, 65–68 10.1007/BF00925738 8350865

[16] Izumi T. and Yamaguchi M. (2004) Overexpression of regucalcin suppresses cell death and apoptosis in cloned rat hepatoma H4-II-E cells induced by lipopolysaccharide, PD 98059, dibucaine, or Bay K 8644. J. Cell. Biochem. 93, 598–608 10.1002/jcb.20214 15378600

[17] Izumi T. and Yamaguchi M. (2004) Overexpression of regucalcin suppresses cell death in cloned rat hepatoma H4-II-E cells induced by tumor necrosis factor-alpha or thapsigargin. J. Cell. Biochem. 92, 296–306 10.1002/jcb.20056 15108356

[18] Nakagawa T. and Yamaguchi M. (2005) Overexpression of regucalcin suppresses apoptotic cell death in cloned normal rat kidney proximal tubular epithelial NRK52E cells: change in apoptosis-related gene expression. J. Cell. Biochem. 96, 1274–1285 10.1002/jcb.20617 16167335

[19] Hughes P.J., McLellan H., Lowes D.A., Kahn S.Z., Bilmen J.G., Tovey S.C. et al. (2000) Estrogenic alkylphenols induce cell death by inhibiting testis endoplasmic reticulum Ca(2+) pumps. Biochem. Biophys. Res. Commun. 277, 568–574 10.1006/bbrc.2000.3710 11061995

[20] Lai P., Yip N.C. and Michelangeli F. (2011) Regucalcin (RGN/SMP30) alters agonist- and thapsigargin-induced cytosolic Ca2+. transients in cells by increasing SERCA Ca(2+)ATPase levels. FEBS Lett. 585, 2291–2294 10.1016/j.febslet.2011.05.058 21684279

[21] Law B., Michelangeli F., Qu Y.Q., Xu S.W., Han Y., Mok S. et al. (2019) Neferine induces autophagy-dependent cell death in apoptosis-resistant cancers via ryanodine receptor and Ca^2+^-dependent mechanism. Sci. Rep. 9, 20034 10.1038/s41598-019-56675-6 31882989 PMC6934498

[22] Lai P. and Michelangeli F. (2009) Changes in expression and activity of the secretory pathway Ca2+ ATPase 1 (SPCA1) in A7r5 vascular smooth muscle cells cultured at different glucose concentrations. Biosci. Rep. 29, 397–404 10.1042/BSR20090058 19527224 PMC2752273

[23] Bolte S. and Cordelières F.P. (2006) A guided tour into subcellular colocalization analysis in light microscopy. J. Microscop. 224, 213–232 10.1111/j.1365-2818.2006.01706.x 17210054

[24] Minotti G., Menna P., Salvatorelli E., Cairo G. and Gianni L. (2004) Anthracyclines: molecular advances and pharmacologic developments in antitumor activity and cardiotoxicity. Pharmacol. Rev. 56, 185–229 10.1124/pr.56.2.6 15169927

[25] Zhang J., Clark J.R. Jr, Herman E.H. and Ferrans V.J. (1996) Doxorubicin-induced apoptosis in spontaneously hypertensive rats: differential effects in heart, kidney and intestine, and inhibition by ICRF-187. J. Mol. Cell Cardiol. 28, 1931–1943 10.1006/jmcc.1996.0186 8899552

[26] Lee T.K., Lau T.C. and Ng I.O. (2002) Doxorubicin-induced apoptosis and chemosensitivity in hepatoma cell lines. Cancer Chemother. Pharmacol. 49, 78–86 10.1007/s00280-001-0376-4 11855756

[27] Rebbaa A., Chou P.M., Emran M. and Mirkin B.L. (2001) Doxorubicin-induced apoptosis in caspase-8-deficient neuroblastoma cells is mediated through direct action on mitochondria. Cancer Chemother. Pharmacol. 48, 423–428 10.1007/s002800100375 11800021

[28] Shin H.J., Kwon H.K., Lee J.H., Gui X., Achek A., Kim J.H. et al. (2015) Doxorubicin-induced necrosis is mediated by poly-(ADP-ribose) polymerase 1 (PARP1) but is independent of p53. Sci. Rep. 5, 15798 10.1038/srep15798 26522181 PMC4629133

[29] Law B., Mok S., Chen J., Michelangeli F., Jiang Z.H., Han Y. et al. (2017) *N*-Desmethyldauricine Induces Autophagic Cell Death in Apoptosis-Defective Cells via Ca^2+^ Mobilization. Front. Pharmacol. 8, 388 10.3389/fphar.2017.00388 28670281 PMC5472688

[30] Koleini N. and Kardami E. (2017) Autophagy and mitophagy in the context of doxorubicin-induced cardiotoxicity. Oncotarget 8, 46663–46680 10.18632/oncotarget.16944 28445146 PMC5542301

[31] Yamaguchi M. (2013) The anti-apoptotic effect of regucalcin is mediated through multi-signaling pathways. Apoptosis 18, 1145–1153 10.1007/s10495-013-0859-x 23670020 PMC3775152

[32] Fukaya Y. and Yamaguchi M. (2005) Overexpression of regucalcin suppresses apoptotic cell death in the cloned rat hepatoma H4-II-E cells induced by a naturally occurring isothiocyanate sulforaphane. Int. J. Mol. Med. 15, 853–857 10.3892/ijmm.15.5.853 15806309

[33] Michelangeli F., Ogunbayo O.A. and Wootton L.L. (2005) A plethora of interacting organellar Ca2+ stores. Curr. Opin. Cell Biol. 17, 135–140 10.1016/j.ceb.2005.01.005 15780589

[34] Takahashi H. and Yamaguchi M. (2000) Stimulatory effect of regucalcin on ATP-dependent Ca(2+) uptake activity in rat liver mitochondria. J. Cell. Biochem. 78, 121–130 10.1002/(SICI)1097-4644(20000701)78:1<121::AID-JCB11>3.0.CO;2-2 10797571

[35] Cappetta D., De Angelis A., Sapio L., Prezioso L., Illiano M., Quaini F. et al. (2017) Oxidative stress and cellular response to doxorubicin: a common factor in the complex milieu of anthracycline cardiotoxicity. Oxid. Med. Cell. Longev. 2017, 1521020 10.1155/2017/1521020 29181122 PMC5664340

[36] Murata T., Yamaguchi M., Kohno S., Takahashi C., Kakimoto M., Sugimura Y. et al. (2018) Regucalcin confers resistance to amyloid-β toxicity in neuronally differentiated PC12 cells. FEBS Open. Bio. 8, 349–360 10.1002/2211-5463.12374 29511612 PMC5832982

[37] Nakagawa T. and Yamaguchi M. (2005) Hormonal regulation on regucalcin mRNA expression in cloned normal rat kidney proximal tubular epithelial NRK52E cells. J. Cell. Biochem. 95, 589–597 10.1002/jcb.20422 15786489

